# Role of magnetic resonance imaging in organ-preserving strategies for the management of patients with rectal cancer

**DOI:** 10.1186/s13244-019-0742-6

**Published:** 2019-05-30

**Authors:** Cinthia D. Ortega, Rodrigo O. Perez

**Affiliations:** 1 School of Medicine, Radiology Department, University of São Paulo, Travessa da Rua Dr. Ovídio Pires de Campos, 75, São Paulo, 05403-010 Brazil; 2 Angelita & Joaquim Gama Institute, São Paulo, Brazil; 3 School of Medicine, Colorectal Surgery Division, University of São Paulo, São Paulo, Brazil; 4 Ludwig Institute for Cancer Research São Paulo Branch, São Paulo, Brazil

**Keywords:** Rectal cancer, Organ preservation, MRI staging

## Abstract

Total mesorectal excision has been the most effective treatment strategy adopted to reduce local recurrence rates among patients with rectal cancer. The morbidity associated with this radical surgical procedure led surgeons to challenge the standard therapy particularly when dealing with superficial lesions or good responders after neoadjuvant radiotherapy, to which radical surgery may be considered overtreatment. In this subset of patients, less invasive procedures in an organ-preserving strategy may result in good oncological and functional outcomes. In order to tailor the most appropriate treatment option, accurate baseline staging and reassessment of tumor response are relevant. MRI is the most robust tool for the precise selection of patients that are candidates for organ preservation; therefore, radiologists must be familiar with the criteria used to guide the management of these patients. The purpose of this article is to review the relevant features that radiologists should know in order to provide valuable information during the multidisciplinary discussion and ultimate management decision.

## Key points

- Baseline MRI staging can identify early lesions that may be appropriate candidates for organ-preserving strategies
- MRI may provide objective information regarding appropriateness of sphincter-preserving procedures
- Good radiological response to neoadjuvant treatment identified by post-treatment MRI may select appropriate candidates to organ-preserving strategies

## Introduction

Total mesorectal excision (TME) with or without neoadjuvant chemoradiation (nCRT) has been the cornerstone of rectal cancer management for the last decades, leading to significant improvement in oncological outcomes by reducing local recurrence rates [1–4]. Unfortunately, substantial risk of perioperative morbidity comes along with this radical procedure [5, 6]. Impairment on quality of life secondary to sexual and urinary dysfunction, poor fecal incontinence scores, low anterior resection syndrome, and the possibility of a permanent stoma are some problems the patients often face after treatment [7, 8].

In the past few years, concerns of the negative effects of treatment led surgeons to challenge the role of the radical surgical procedure for early-stage tumors or for good responders after nCRT [9–12]. As an alternative to TME, superficial tumors with low risk of lymph node dissemination could be managed by local excision with no radiotherapy [13] or after nCRT in the setting of good clinical response [12, 14]. In very selected patients who achieve a complete clinical response after nCRT, a strict surveillance program without immediate surgery (“watch-and-wait”) may lead to good oncological and functional outcomes [9, 10].

**Fig. 1:**
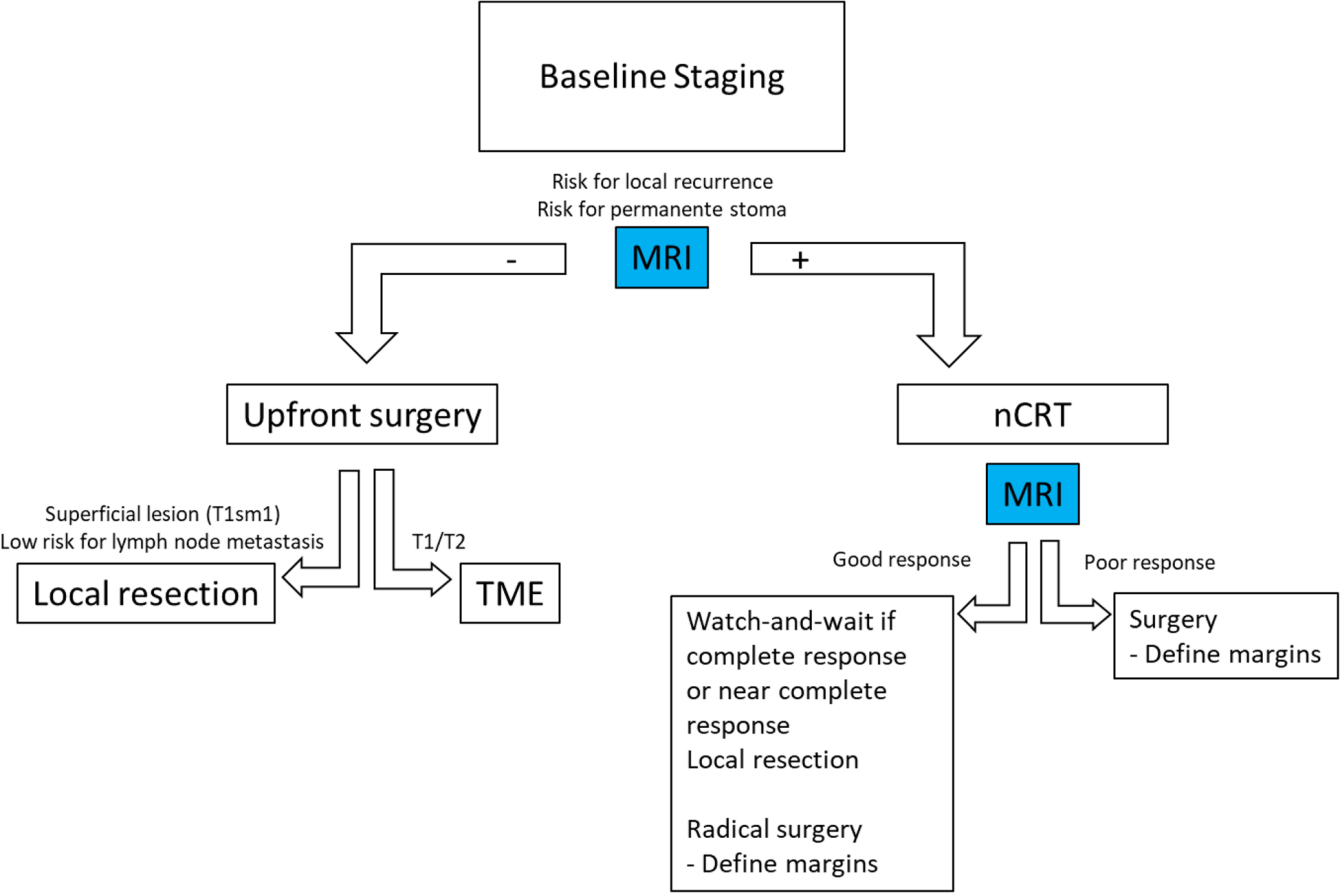
Roles of MRI in staging patients with rectal cancer

Magnetic resonance (MRI) with high-resolution T2-weighted images plays a key role in the decision management strategy of these patients. First, MR has become a routine practice for primary staging of rectal cancer [15, 16]. Critical features at baseline MR staging will aid in the appropriate selection of candidates to be managed by upfront local excision, TME, or by preoperative neoadjuvant therapy [17, 18]. Among patients undergoing the latter strategy, evaluation of response by MR restaging may be critical in assisting surgeons planning a clear-margin resection [19–22]. In addition, MRI restaging may also play a role in selecting ideal candidates for less radical approaches including local excision or watch-and-wait (Fig. 1). The present review details the use of MRI in the optimal selection of definitive surgical or non-surgical treatment for patients with rectal cancer. The topic is particularly relevant when it comes to rectal tumors lying below the peritoneal reflection. In this setting, surgical treatment is significantly more complex and associated with higher rates of functional consequences. Here, we detail a review of pivotal staging information that radiologists must be aware of to provide valuable data for multidisciplinary discussion during the decision management process of these patients.

## Initial staging

Initial locoregional rectal cancer staging aims to select patients with either high risk for local recurrence features or high risk for a permanent stoma. Both groups of patients may benefit from nCRT. In contrast, patients with early tumors can be safely managed by upfront surgery (TME). MRI is the most robust tool to detect these relevant risk factors, and proper scans are capable of detecting poor-prognosis criteria that should guide the management decision of these patients [17, 23]. MRI should be able to identify: involvement or threatening of the mesorectal fascia, T3 lesions that extend more than 5 mm beyond the muscularis propria layer, the presence of ≥ 3 metastatic mesorectal lymph nodes (N2), extramural vascular invasion (EMVI), and pelvic side lymph nodes.

**Fig. 2:**
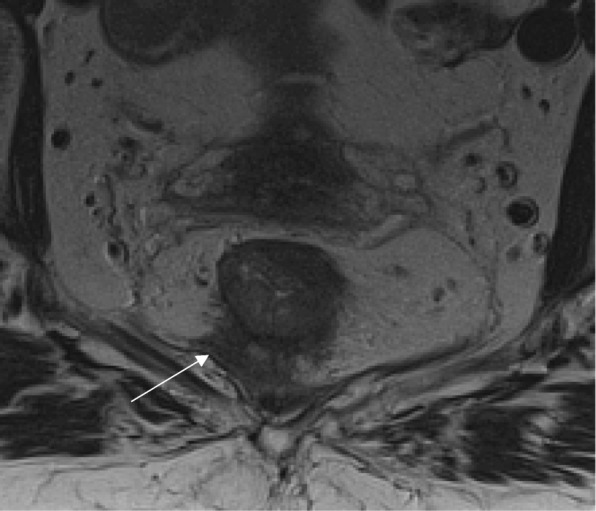
Mesorectal fascia status. High-resolution axial T2WI shows a semiannular tumor infiltrating the mesorectal fat and threatening the mesorectal fascia (arrow)

**Fig. 3:**
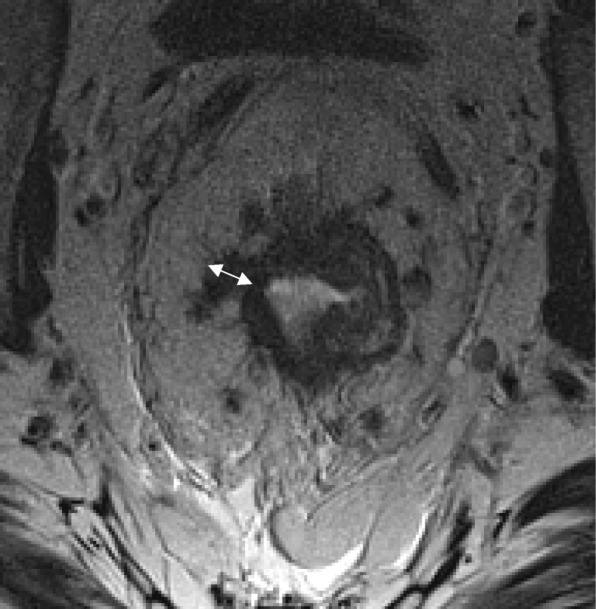
T3 substage. High-resolution axial T2WI shows an annular tumor that extends beyond the muscularis propria. The measurement of the 9-mm spread was taken at the infiltrative border of the lesion at 9 o’clock (arrow)

**Fig. 4:**
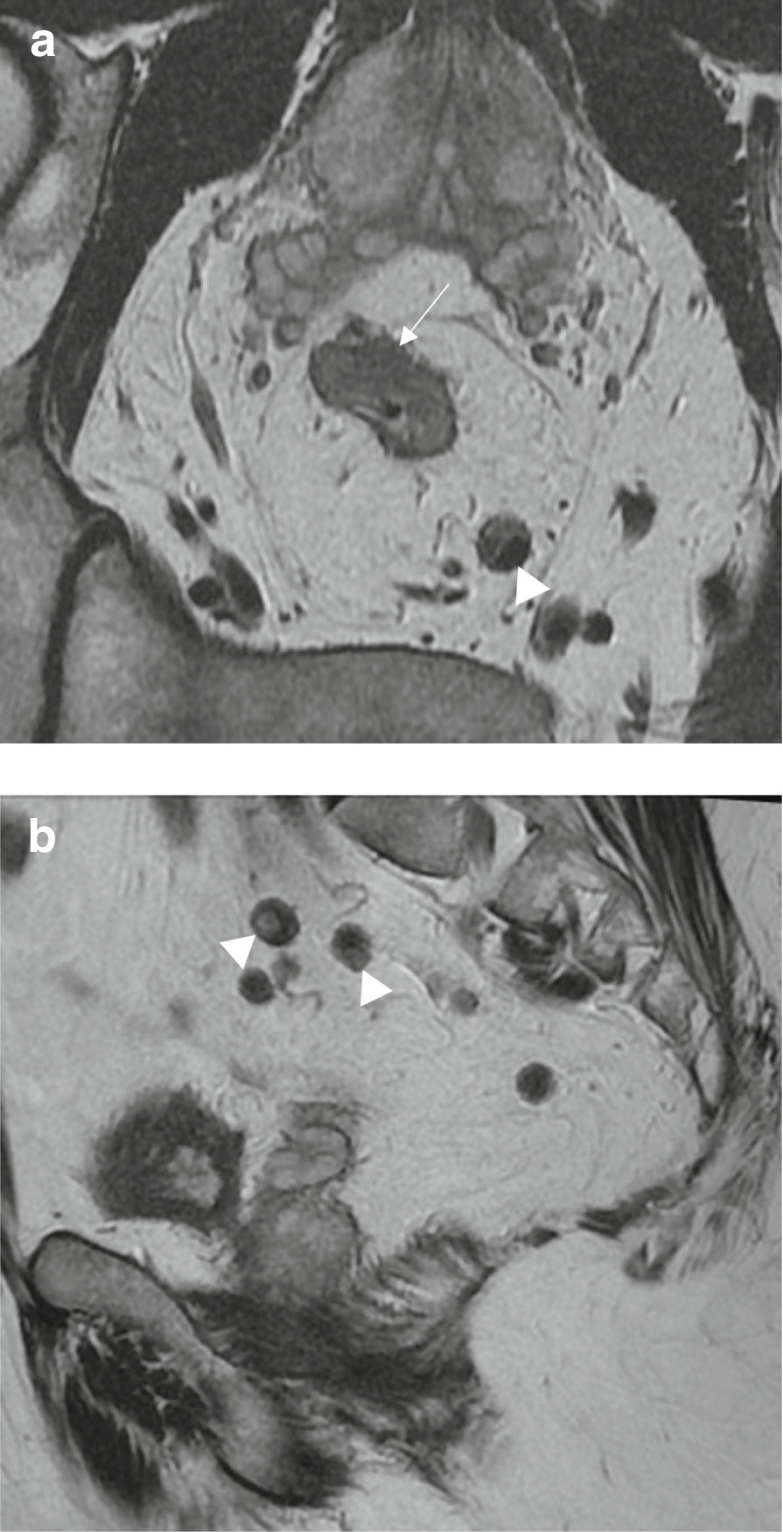
Lymph node staging criteria. **a** High-resolution axial T2WI shows a semiannular anterior tumor (arrow) and a mesorectal involved lymph node showing mixed signal intensity (arrowhead). **b** High-resolution sagittal T2WI shows other involved lymph nodes with mixed signal intensity (arrowheads)

**Fig. 5:**
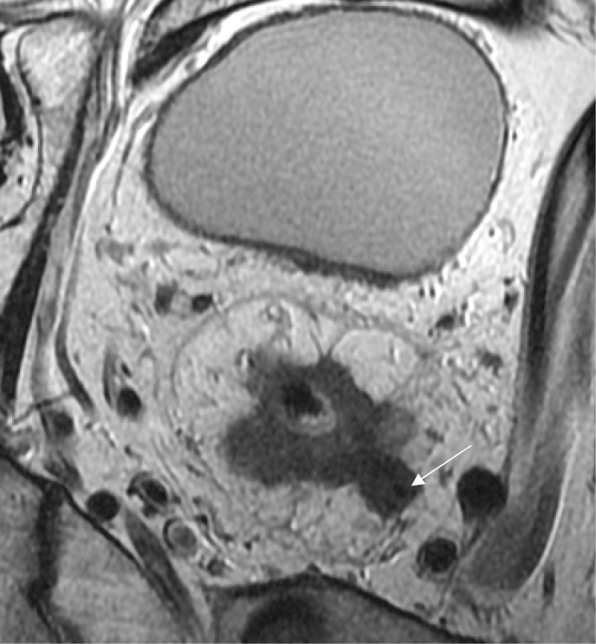
Extramural vascular invasion. High-resolution axial T2WI shows an annular tumor infiltrating the mesorectal fat. A mesorectal vessel flow void (arrow) is involved by nodular extension of tumoral signal intensity beyond the muscularis propria

As previously described, baseline staging of rectal tumors should include [15, 16, 23, 24]:

- Evaluation of the mesorectal fascia: if tumor, EMVI or deposits are within 1 mm of the mesorectal fascia, the latter should be considered involved (Fig. 2)
- T3 substage: T3 tumors should be subclassified according to the maximal depth of spread into the mesorectal fat. That should be measured from the edge of the outermost muscularis propria layer (Fig. 3)
- Lymph node staging: morphological criteria used for mesorectal or pelvic side node positivity include border irregularity or mixed signal intensity (Fig. 4)
- Extramural venous invasion: it is depicted as tumor signal into or along mesorectal vessels, which may lose the characteristic flow-void on T2WI, that show enlargement or contour irregularity (Fig. 5)

When none of these known risk factors are present, patients can be managed by TME surgery alone provided there is good surgical technique and an intact TME specimen [18].

### Surgical alternatives: TME or local excision

TME is a surgical procedure that completely removes the rectum harboring the primary cancer along with the mesorectal fat containing lymph nodes, vessels, and tumor deposits [25, 26]. By removing the entire and intact mesorectum with clear circumferential resection margins (CRM), the risk of local recurrence decreases even in patients with nodal or extramural vascular spread [27, 28].

**Fig. 6:**
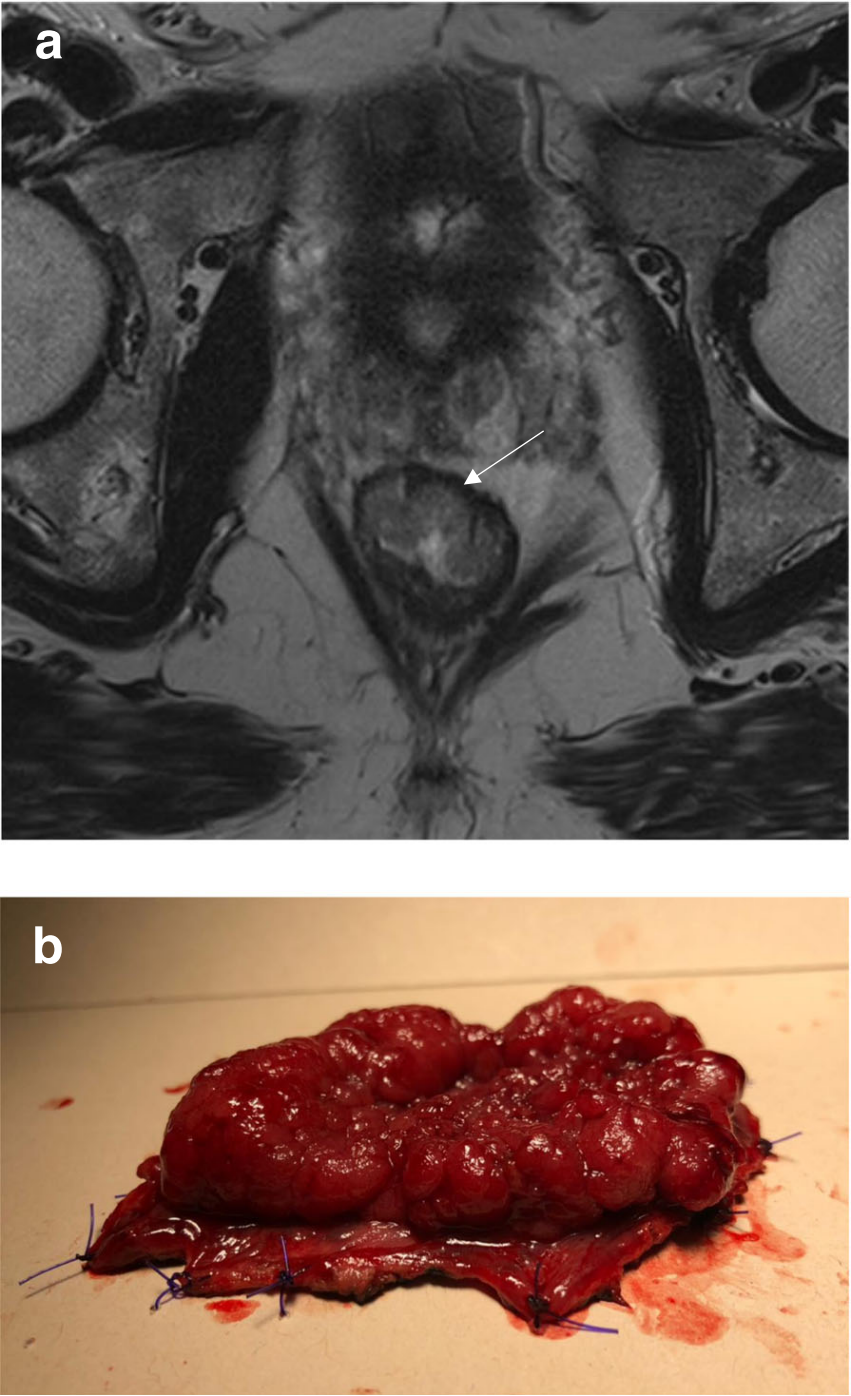
Early rectal cancer staging. **a** High-resolution axial T2WI shows a semiannular low lying lesion extending from 10 to 3 o’clock confined to the rectal wall with no signs of invasion of the muscularis propria (arrow). The lesion was staged as mrT1N0, and local excision was performed. **b** Surgical specimen. The resected specimen after local excision. Histopathological analysis diagnosed adenoma

In contrast, transanal local excision is a procedure that exclusively removes the primary tumor by a full-thickness incision of the rectal wall in the area bearing the primary tumor. The procedure is currently more frequently performed using modern endoscopic platforms allowing for higher rates of margin negative and non-fragmentation of the specimen [29]. During local excision, there is no formal removal of draining lymph nodes, even though eventually a few lymph nodes may be recovered from the resected specimen present in the surrounding perirectal fat [30]. Therefore, only early rectal lesions confined to the bowel wall and showing minimal risk for lymph node metastases are appropriate candidates to local excision as a definitive and curative procedure. The greatest advantage of this procedure is the avoidance of the potential mortality and morbidity of radical surgery while maintaining acceptable oncological outcomes [13, 29] (Fig. 6).

#### Candidates for local excision

Here, the primary purpose of staging is the identification of unfavorable features in order to rule out a local procedure without formal mesorectal excision. Primary depth of tumor infiltration (T-stage), submucosal lymphovascular invasion, budding and tumor grade are known histopathologic predictors of mesorectal nodal metastases (pN+) [13, 31]. Apart from histological criteria, available only after resection, imaging may provide the identification of features that may also predict a poor outcome after local excision. The presence of involved lymph nodes, extramural vascular invasion, and tumors invading beyond the muscularis propria (≥ pT3) are all clear contraindication for a local procedure as a definitive treatment in rectal cancer patients with curative intent [15, 32].

The risk of lymph node involvement among pT2 lesions is also quite considerable, so most T2 lesions should be preferably not managed by local excision alone as a definitive curative intent treatment modality [31, 33]. There is some data supporting the use of local excision followed by adjuvant chemoradiation in small and superficial pT2 cancers [14]. However, TME offers the best chances of cure among most of these patients.

Due to the absence or low risk of pN+, local excision is the preferred treatment alternative for the management of adenomas, in situ adenocarcinoma, and select T1 lesions. Sessile T1 lesions can be subclassified using the Kikuchi submucosal staging system into three levels: sm1 lesions infiltrate up to the upper third of the submucosa, sm2 up to the middle third, and sm3 up to the lower third [34]. Alternatively, precise measurement of depth invasion may also provide accurate identification of patients at lower risk for pN+. Patients with ≤ 1000 μM of depth of tumor invasion (sm1) are considered appropriate candidates for local excision alone [35]. Risk stratification shows that T1sm3 lesions are associated with nodal spread at almost similar rates to T2 lesions. Therefore, T1sm1 tumors are the ones associated with lower risk of nodal metastases and recurrence and that can be safely managed by local excision alone [36].

#### Staging

Clinical selection of this specific subgroup of early lesions is challenging. Available options to staging early rectal tumors are MRI, endorectal ultrasound, and magnifying colonoscopy or image-enhanced endoscopy [37, 38]. Briefly, endorectal ultrasound is capable of distinguishing between T1 and T2 lesions with good overall accuracy, and the main limitations include large lesions, polyps, or lesions lying on the upper third of the rectum. Endoscopic evaluation of the pit pattern of rectal lesions is one of the most accurate methods to preoperatively distinguish between a benign or a malignant lesion. In addition, it provides information regarding deep submucosal invasion in superficial tumors [38]. Precise endoscopic findings and patterns associated with distinct histological findings are provided elsewhere and are beyond the scope of this review.

**Fig. 7:**
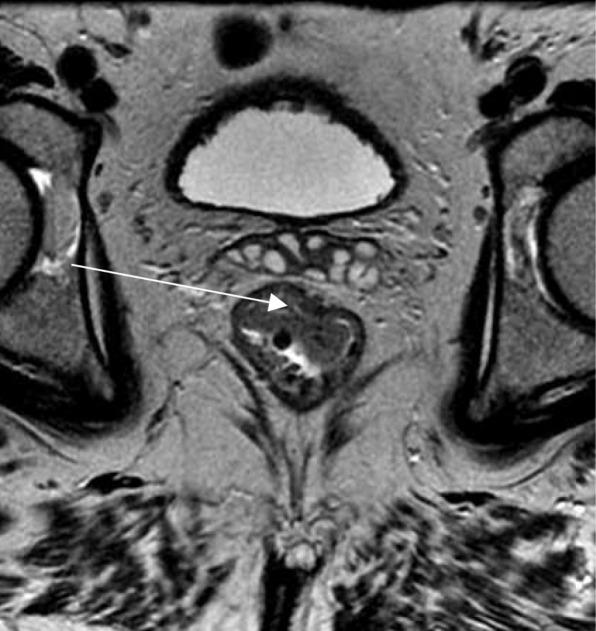
The high-signal intensity of the submucosa. High-resolution axial T2WI shows a semiannular low-lying lesion extending from 10 to 1 o’clock confined to the rectal wall with no signs of invasion of the muscularis propria and preserved high-signal intensity of the submucosal layer (arrow). The lesion was staged as mrT1N0

MRI may help evaluate the presence of extramural disease with good specificity. It is the most accurate method to evaluate tumor extension beyond the muscularis propria and extramural vascular invasion and shows good positive predictive values in assessing mesorectal nodes [17, 39, 40]; however, distinguishing between T1 and T2 lesions is sometimes not straightforward. Considering that T1 classification is not enough to guide treatment, subclassification of T1 is required. It has been shown that a visible measurement of 1 mm or more of preserved high-signal intensity of the submucosa (Fig. 7) can be used as a predictor of partial submucosal invasion [32]. In this framework, using MRI to distinguish between T1 and T2 lesions may be challenging, but distinguishing between early T1sm1–2 prone to local excision and T1sm3/T2 lesions that are not ideal candidates for local resection may be more accurate.

Overall, the roles of MRI when evaluating early rectal tumors if local resection is considered are:

1. Detection of any unsuspected mesorectal disease—positive nodes and/or vascular invasion
2. Evaluation of T-stage—submucosal high signal intensity preservation to exclude T1sm3/T2 lesions

If there is neither mesorectal disease nor deep infiltration of the rectal wall seen on the MRI, local excision can be considered an appropriate alternative. Here, local excision may act as an excisional biopsy. If histopathology confirms preoperative staging (< pT1sm3), local resection may be considered curative. However, if histopathology shows ≥ pT1sm3, lymphovascular invasion, budding, or other unfavorable features, additional treatment after local excision may still be necessary [13, 33]. Alternatives for additional treatment may include completion of TME or adjuvant/postoperative chemoradiation (CRT) [33, 41, 42].

### Sphincter preservation

**Fig. 8:**
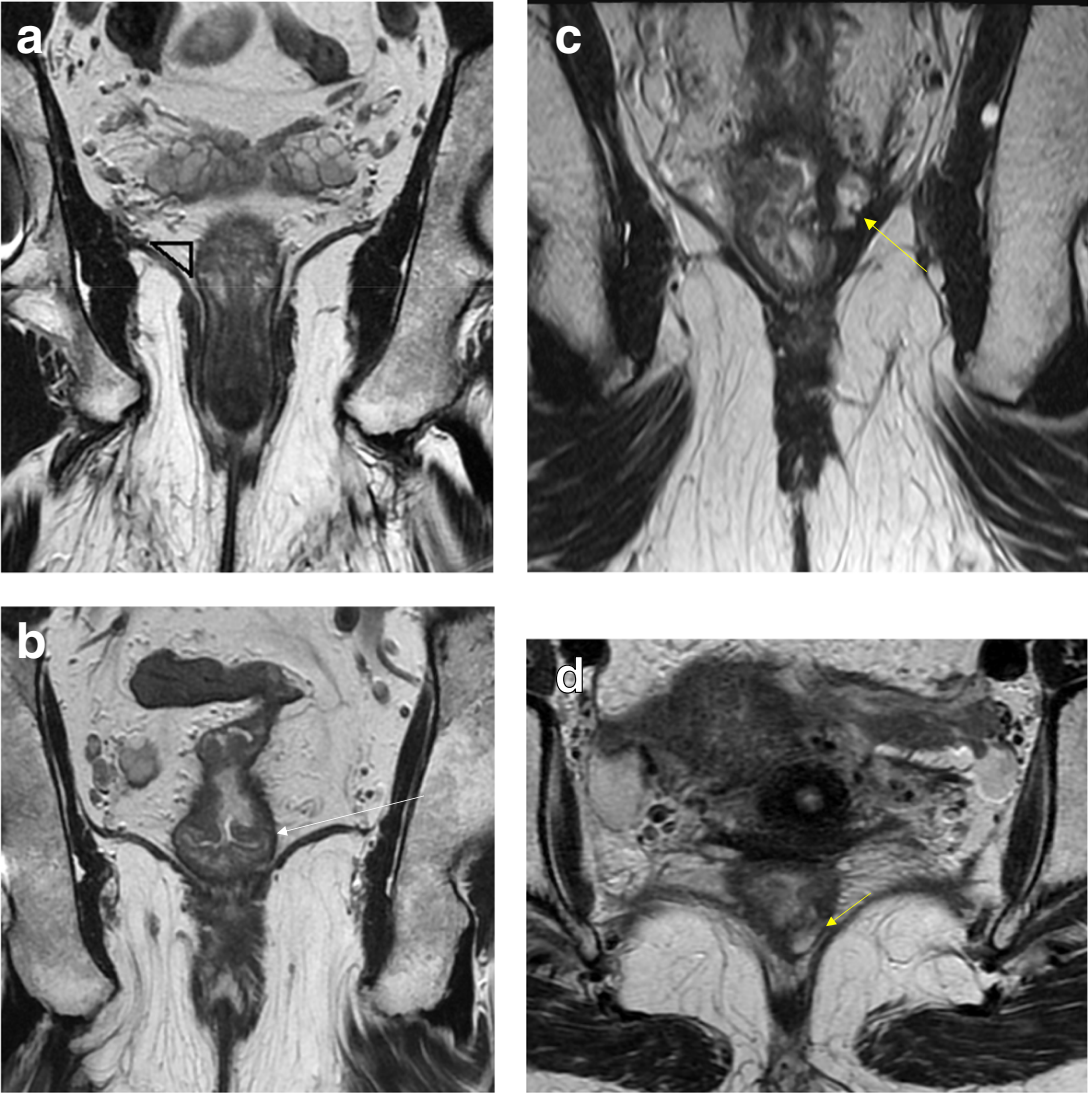
The assessment of the intersphincteric plane safety. **a** Coronal T2WI shows the normal anal canal anatomy. The distal tapering of the mesorectal fat is shown by the black triangle that delineates the fat content on the upper portion or the dissection plane when low anterior resection is performed. **b** Coronal T2WI shows the distal edge of the lesion (arrow) above the intersphincteric plane. Low anterior resection is possible with no risk of margin positivity at this level. **c** Coronal T2WI shows the distal edge of the lesion lying in the plane of the levators. There is lateral spread with mucinous content on the left, threatening the intersphincteric plane (arrow). **d** High-resolution axial T2WI shows a residual low-lying lesion partially invading the muscularis propria (arrow). Intersphincteric resection would be safely performed. A clear resection margin was achieved

According to the location and staging, low rectal tumors that undergo surgery may require abdominoperineal resection (APR) with a permanent stoma or coloanal anastomosis with occasionally poor functional outcomes [8, 32, 43]. Traditionally, surgeons would make the decision between an APR or a restorative procedure with sphincter preservation based on the distance of the tumor from the sphincter complex and preoperative function. Patients with already preoperative incontinence were obviously considered for APR. However, from an oncological standpoint, the ability to achieve a safe distal margin with no direct invasion of the sphincters was considered the sole required condition for a sphincter-saving procedure. This assessment was almost universally done through simple clinical or digital rectal examination. Currently, however, MR offers the opportunity to assess more accurately the integrity of the surgical plane required for organ preservation [44, 45]. During TME with sphincter preservation, the surgeon will have to dissect between the mesorectal plane and the levator muscles. As the plane progresses distally, the mesorectal plane tapers and becomes closer to the levator ani muscles. The integrity of such plane is crucial for obtaining an R0 mesorectal plane dissection with sphincter preservation. This is accurately provided by MR [46] (Fig. 8). A recent classification of distal rectal tumors provided by MRI and clinical assessment describes four types and their preferred surgical management strategies. The first I–III subtypes (supra-anal, juxta-anal, and intra-anal) may provide the opportunity for sphincter preservation. Type IV (transanal) is best suited for APR [45].

### Organ preservation

nCRT has been used with the purpose of avoiding local recurrence by downstaging and downsizing locally advanced rectal tumors resulting in increased rates of clear resection margins [1, 2]. A proportion of these patients will achieve complete pathological response (pCR) with no tumor seen in the surgical specimen after nCRT. This has raised the question to whether surgery was necessary or otherwise resulted in overtreatment of this subgroup of patients, particularly when a permanent stoma is required [9]. For these reasons, patients who achieve complete clinical response (cCR), with no visible residual tumor when assessed by digital rectal examination or endoscopy, have been managed without immediate surgery [47, 48]. A significant proportion of patients that achieve cCR may reflect complete pathological response (pCR). Deferring ultimate surgical management of these patients with cCR or apparent complete pathological response is currently considered an acceptable alternative in order to avoid potentially unnecessary morbidity and mortality of radical surgery and still achieve excellent/similar oncological outcomes [47, 49–51].

Several features may influence the development of a cCR including treatment-related and tumor-related features. Baseline staging features have been considered a predictor of long-term outcome after organ preservation. Depth of tumor penetration seems to correlate directly with the risk of developing tumor regrowth after initial “apparent” complete clinical response and non-operative management [52, 53]. The chances of successful organ preservation are higher for early baseline tumors (mrT2) and lower for more advanced baseline lesions (mrT3 or mrT4) [11, 53]. In contrast, nodal disease at baseline has not been associated with lower rates of successful organ preservation [54].

In this context, MRI plays a role in the selection process of patients who are ideal candidates to this approach. Tumors with no adverse features/“good tumors” may be managed by upfront surgery without neoadjuvant CRT, (T2 or T3a/bN0–1 tumors), whenever the surgical alternative would otherwise require an APR or an ultra-low anterior resection (associated with poor anorectal function) [55]. This particular subgroup of patients could be staged and guided to neoadjuvant therapy as an attempt to achieve cCR and undergo a non-operative management pathway. Here, the purpose of nCRT is not to decrease the rates of a positive margin, but to achieve primary tumor regression, possibly avoid surgery and to improve functional outcomes. Although early cT3 lesions may also achieve cCR, retrospective analysis indicates that the risk of early regrowths within the first year of follow-up is increased when compared to cT2 lesions [52]. Therefore, distinguishing between cT2 vs cT3a low rectal tumors may be relevant if organ preservation is considered.

In addition, when chemoradiation is considered for the purpose of achieving cCR, treatment-related features may become relevant. There is also data to suggest that higher radiation therapy doses (≥ 50.4 Gy) and additional chemotherapy cycles (consolidation chemotherapy) may increase the chances of primary tumor regression [56, 57]. The benefits of more aggressive treatment here seem to be associated with baseline staging features [11, 58]. Therefore, primary staging and chances of achieving cCR after treatment may aid multidisciplinary team decision regarding optimal radiation therapy and chemotherapy regimens taking the possibility of organ preservation into account.

**Fig. 9:**
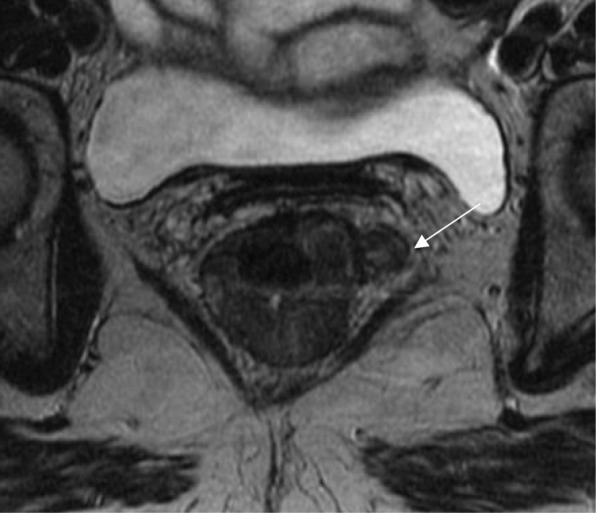
Low rectal cancer staging. High-resolution axial T2WI shows a semiannular lying lesion with tumor deposit extending to the mesorectal fat and threatening the mesorectal fascia (arrow). The lesion was staged as mrT3 with positive mesorectal fascia and positive EMVI. The patient was sent to nCRT

**Fig. 10:**
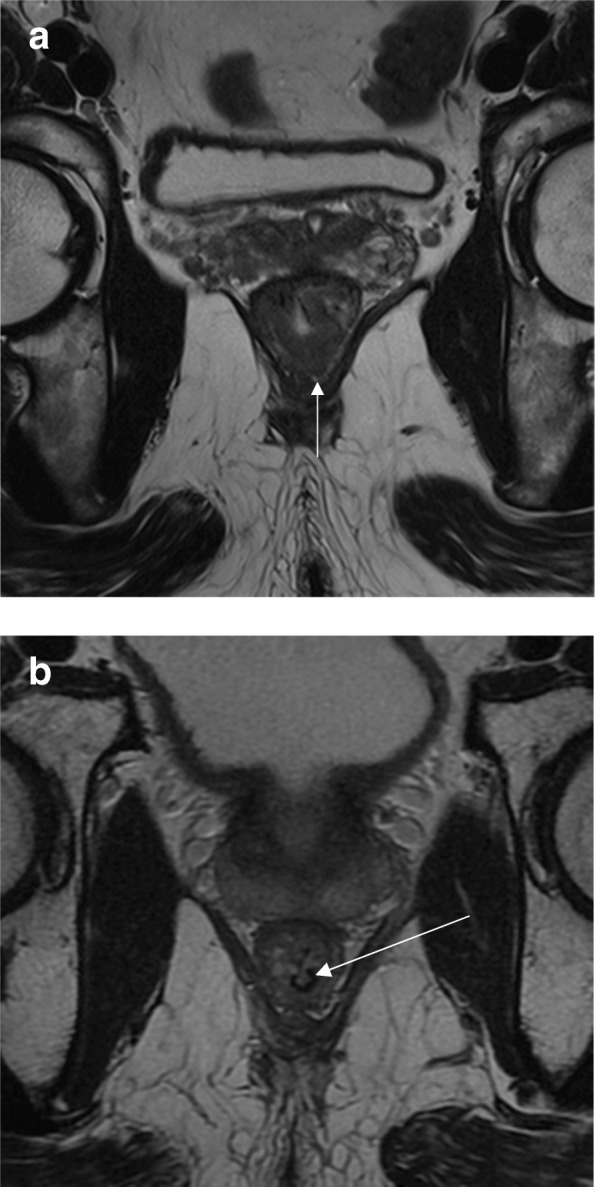
Early low rectal cancer staging. **a** High-resolution axial T2WI baseline staging shows a low-lying posterior lesion extending from 2 to 10 o’clock confined to the rectal wall with no signs of intersphincteric plane involvement (arrow). The lesion was staged as mrT2N0, and the patient was referred to nCRT in an attempt to achieve cCR. **b** Restaging 12 weeks after neoadjuvant CRT shows a low SI scar (arrow). A watch-and-wait approach was attempted. Two years of follow-up show no regrowth

In summary, important information provided by MRI when evaluating low rectal tumors include:

1. Staging and defining risks of a positive CRM to guide nCRT (Fig. 9)
2. Staging early lesions not at risk of positive CRM, but that may benefit from nCRT tailored to achieve cCR (Fig. 10)
3. Determination of intersphincteric plane invasion, suggesting the need for APR

## Restaging after radiotherapy

Response to nCRT can be assessed according to institutional protocols, in general varying from 6 to 12 weeks after the end of radiotherapy [59]. Poor responders usually undergo surgery, and the main role of MRI in this situation is road-mapping the resection planes so that the decision of the most appropriate surgical approach capable of achieving a clear-margin resection is taken [19, 21, 22, 60].

**Fig. 11:**
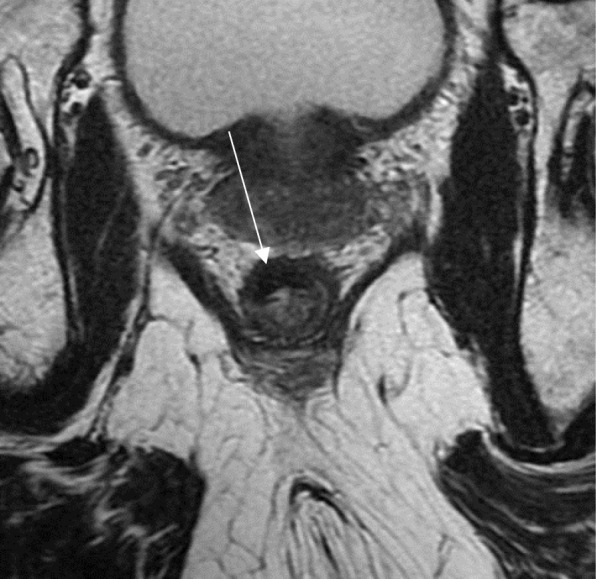
Watch-and-wait approach. High-resolution T2WI restaging shows a low signal intensity fibrotic full-thickness scar extending from 9 to 12 o’clock (arrow). Four-year follow-up showed no regrowth

In contrast, the population showing a good response to nCRT evaluated by digital exam, endoscopy, and MRI criteria might be selected to less radical options [48, 61–63]. Good responders can be selected to local excision or to clinical and imaging reassessments (“watch-and-wait”) in a non-operative approach (Fig. 11). In this latter approach, no immediate surgery is performed until the presence of viable tumor is confirmed by either digital rectum exam (DRE), endoscopic, or imaging evidence of tumor regrowth [64–66]. If regrowth is detected, then radical surgery may be unavoidable.

**Fig. 12:**
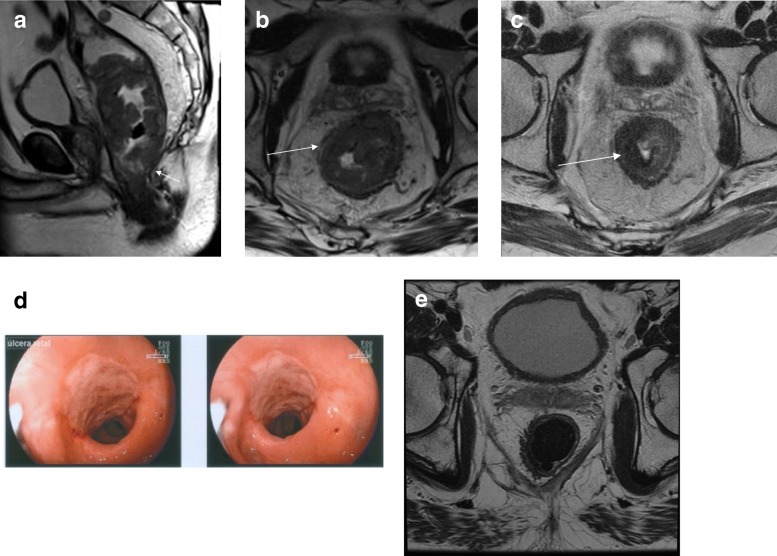
Long-time intervals of reassessment. **a** Sagittal T2WI shows the baseline staging of a low rectal tumor with the distal margin lying below the intersphincteric plane (arrow). **b** Axial T2WI shows the semiannular lesion (arrow). **c** Restaging after 10 weeks showed a good response with predominant low-signal intensity within the lesion (arrow). **d** Proctoscopy showing good but incomplete response with residual ulceration. **e** Reassessment after longer intervals showed evidence of ongoing response to CRT. Follow-up exams after 22 weeks revealed findings consistent with complete clinical response

When the watch-and-wait strategy was first proposed by Habr-Gama et al., only patients that achieved complete clinical response were eligible, meaning that strict criteria of endoscopic and clinical response were to be followed without immediate radical surgery [9, 48]. This practice, initially performed by a single center in Brazil, is now performed in several centers, which allows more data to be published [47, 53, 67, 68]. As data related to the follow-up of patients has increased, it is now proposed that near-complete responders may also be candidates to deferral of surgery [66, 69]. This has been supported by the observation that a significant proportion of patients only achieve cCR (strict criteria) after longer than 8–12 weeks intervals from radiation completion (Fig. 12) [70].

### Tumor regression grade (TRG), diffusion-weighted MR imaging (DWI), and staging

Lesions with complete response after radiotherapy show either a normal rectal wall, whitening of the mucosa with or without some telangiectasia when clinically assessed by endoscopy or DRE [48]. Residual mucosal abnormalities may be present even in pCR, making endoscopic assessment alone insufficient to select possible complete responders properly [71].

When it comes to radiological assessment, good responders show T2WI low signal intensity fibrosis replacing the tumor within the rectal wall or even beyond [49, 60, 72]. The amount of fibrosis can be estimated by MRI through the TRG classification. Results of the MERCURY trial show good correlation between mrTRG and oncologic outcomes; therefore, the amount of fibrosis seen on MRI is a biomarker of response and better oncological results after radical surgery [73]. Good responders show predominant sign of fibrosis on T2WI MRI, with either no signal of tumor or minimal findings. Those lesions are classified as TRG1–2 [49].

DWI is a functional technique used to detect tissues with increased cellularity, which causes restriction to the diffusion of water molecules, resulting in high-signal intensity seen by this sequence. Thus, DWI is useful to detect residual cancer tissue after nCRT and may add information regarding tumor response by increasing the confidence rate of radiologists and increasing interobserver agreement rates [74].

Restaging MRI has a role in detecting those good responders and is fundamental in detecting extraluminal disease not assessed or accessible by endoscopy or DRE—lymph nodes, extramural venous invasion, tumor deposits, lateral pelvic side nodes, peritoneal, or other metastatic diseases [64].

The combination of favorable clinical, endoscopic, and imaging criteria may suggest that good responders should be reassessed and not be immediately sent to surgery—or even managed by transanal local excision—if organ-preserving strategy is pursued, especially when it comes to low-lying tumors that require a permanent stoma or ultra-low anastomoses with poor functional outcomes.

### Interval of reassessment

The optimal timing of reassessment after the end of radiotherapy is still controversial. Retrospective data show that long intervals between the end of nCRT and surgery may result in increased proportions of pCR [75]. When the tumor is reassessed 6–12 weeks after the end of radiotherapy, cCR may already have occurred in some patients. In a proportion of patients, a near cCR may be seen with still some irregularity at DRE, a small residual ulcer or irregular wall thickening at endoscopy, or minimal tumor sign within the fibrosis [69, 71].

If surgical resection is performed at that time, residual tumor may be present within the specimen. Nevertheless, tumor regression may continue beyond 12 weeks, making it impossible to be sure whether further response to radiotherapy was still ongoing. Waiting longer than the 12-week interval to reassess response may result in a larger proportion of patients who achieve a cCR [10, 76].

A prospective multicentric randomized trial that compared the rates of pCR when patients were evaluated either 7 or 11 weeks after the end of nCRT failed to show difference between the intervals; moreover, additional morbidity was seen on the 11-week group [59]. The results of additional randomized trials evaluating tumor response after 6 or 12 weeks are awaited to further clarify the optimal timing for assessment of these patients.

### Follow-up and regrowth

**Fig. 13:**
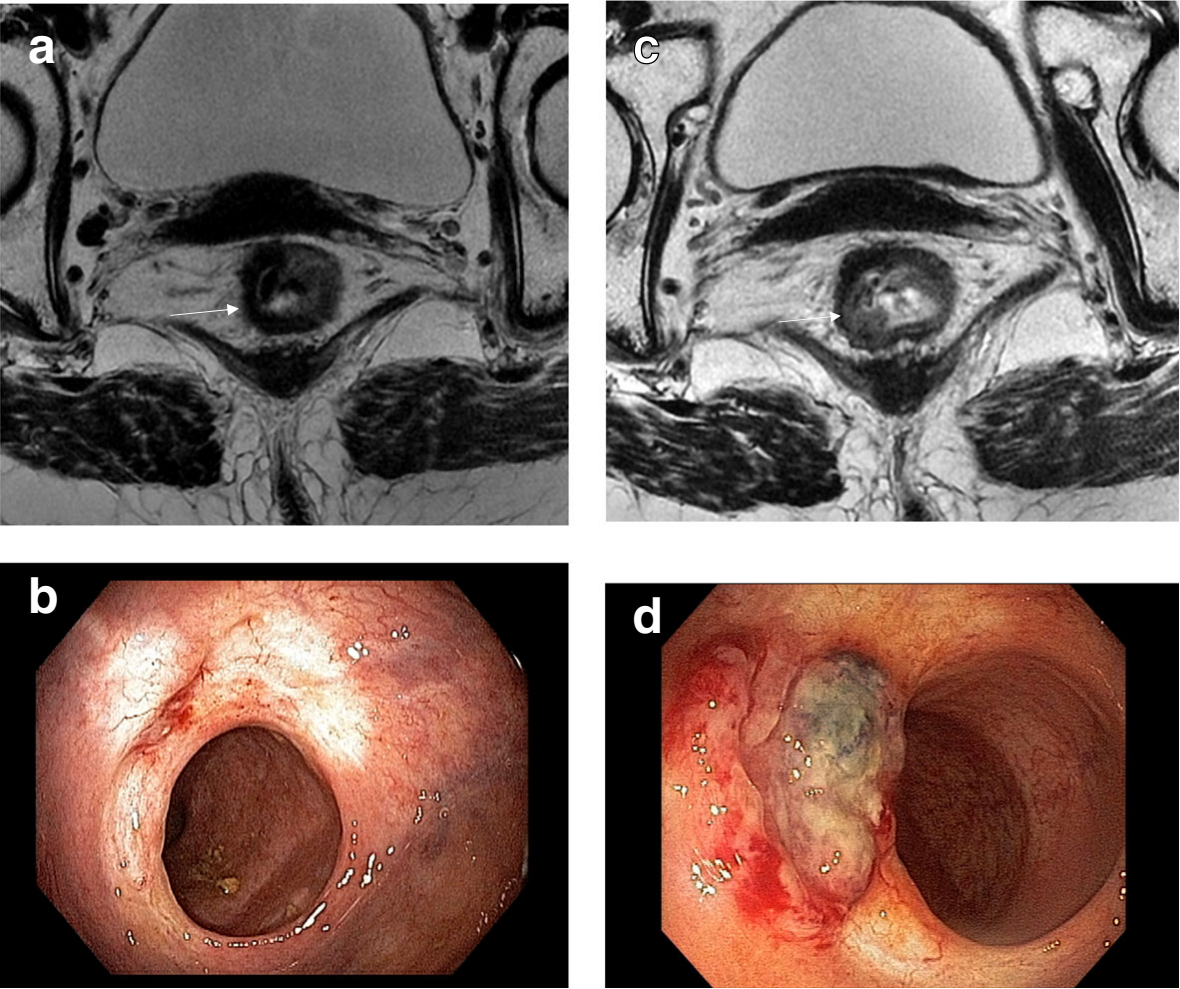
Luminal regrowth detected by MRI and endoscopy. **a** High-resolution T2WI restaging after 8 weeks of completion of CRT shows a low signal intensity fibrotic full-thickness scar extending from 5 to 12 o’clock (arrow). The patient was referred to watch-and-wait. **b** The endoscopic view of the treated lesion after 8 weeks of completing CRT shows whitening of the mucosa with some telangiectasia and no significant residual ulceration. **c** Follow-up after 16 months of completion of CRT shows increased signal intensity where the scar was previously seen from 6 to 9 o’clock (arrow). The patient was referred to salvage resection with total mesorectal excision. A clear margin resection was performed. **d** The endoscopic view of the treated lesion after 16 months of completing CRT shows evident tumor regrowth

**Fig. 14:**
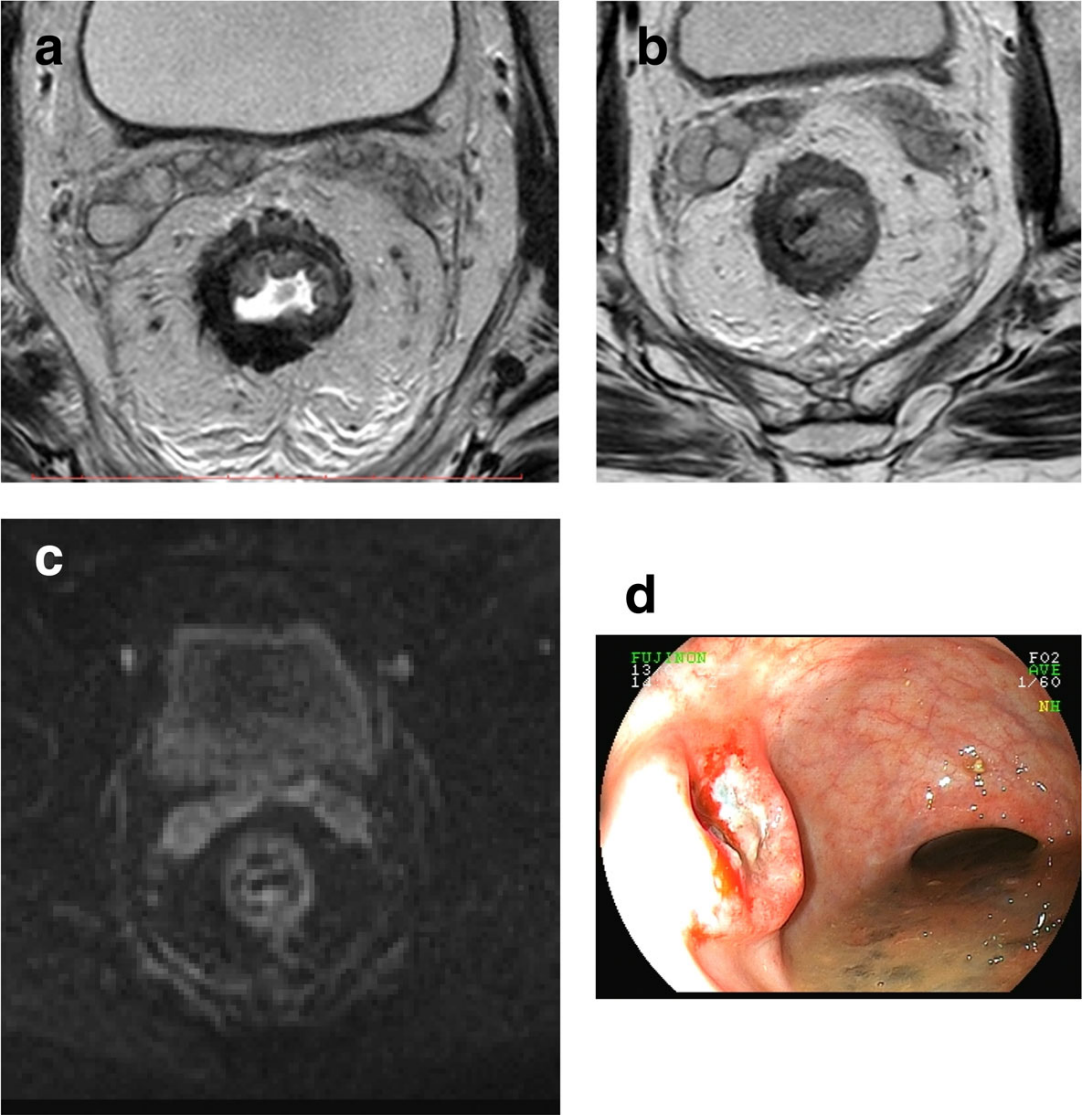
Luminal regrowth detected exclusively by endoscopy. Images show a clear endoscopic view of a regrowth following initial complete clinical response with no relevant radiological findings. **a** High-resolution T2WI restaging axial image 16 weeks after neoadjuvant therapy shows a low signal intensity scar from 5 to 9 o’clock. Watch-and-wait was offered. **b** Follow-up exam after 12 months showed less extensive, from 7 to 10 o’clock persistent low signal intensity scar with no signs of regrowth. **c** DWI (*B* = 700) was unremarkable. **d** Endoscopy showed obvious regrowth at 12 months and the patient was sent to salvage resection. Total mesorectal excision was performed; ypT2N0 with free margins was the final result

**Fig. 15:**
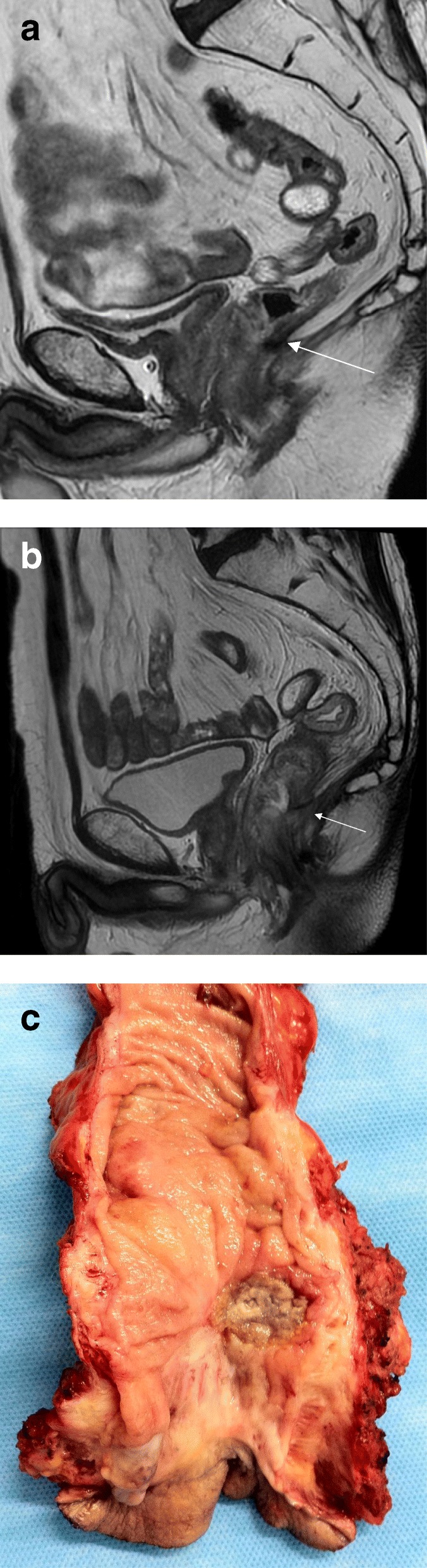
Low rectal lesion with luminal and mesorectal regrowth. **a** High-resolution sagittal T2WI shows the posterior low signal intensity scar (arrow). **b** Follow-up exam after 5 months showed increased signal intensity within the scar and extension to the intersphincteric plane (arrow). **c** The specimen shows the luminal lesion close to the anal verge. Extralevator APR was required and performed with coccygeal resection. Final pathological examination confirmed ypT3N0 with clear (4 mm) circumferential resection margins (R0)

**Fig. 16:**
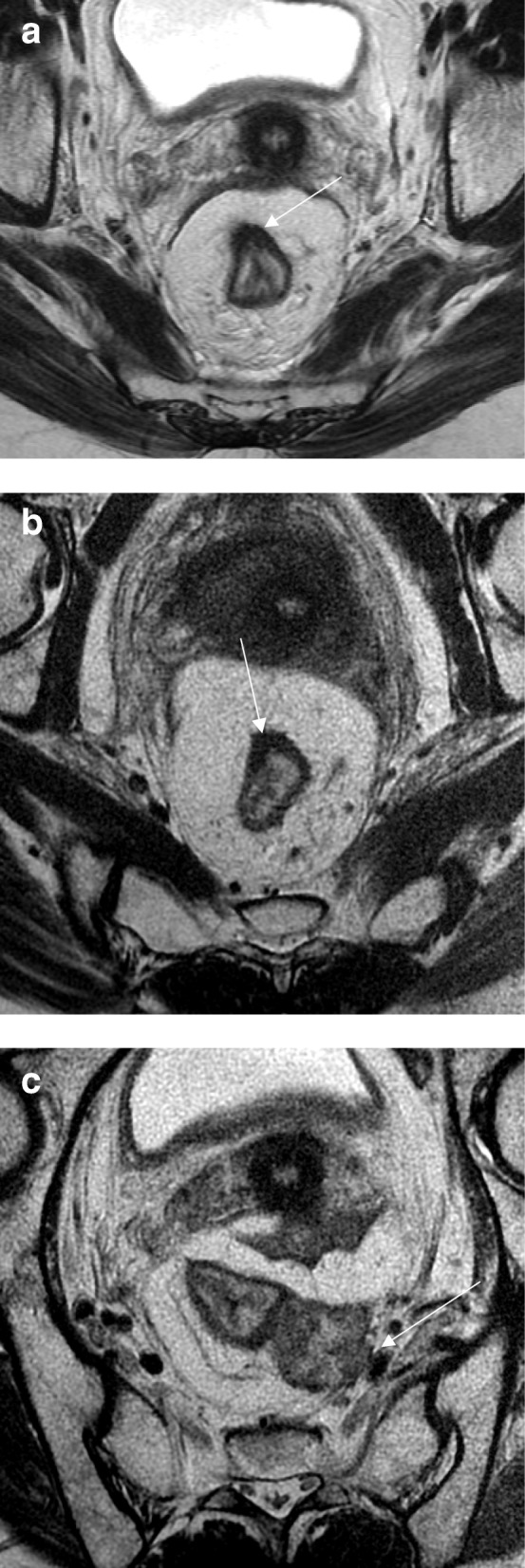
Mesorectal regrowth. **a** Axial T2WI shows an anterior low signal intensity scar (arrow). No mesorectal disease was visible. **b** Follow-up exam after 12 months showed an unchanged low signal intensity scar within the anterior wall of the rectum (arrow). **c** A large mesorectal lymph node or deposit was visible, threatening the mesorectal fascia (arrow). Total mesorectal excision was performed (ypT0N1) with a clear (2 mm) circumferential resection margin

The purpose of follow-up or surveillance studies is to detect possible tumor regrowths after initial “apparent” complete clinical response. Most of these regrowths represent viable tumor that repopulates the area of the primary tumor within the rectal wall. The interval of required surveillance exams remains unclear. Since most regrowths occur during the first 2 years of follow-up, imaging evaluations should be more frequent during this period [53]. Endoscopy and DRE evaluate luminal regrowths. MRI is helpful to detect regrowths within the rectal wall deeper than the mucosa or exclusively (less frequent) extraluminal disease—mesorectal or extramesorectal (Figs. 13, 14, 15, 16).

Most regrowths are luminal, detected by endoscopy or DRE. MRI may also detect luminal regrowths as low signal intensity scar changes and tumor intermediate signal intensity appears. Comparison between follow-up exams is useful as changes of signal intensity within the scar may be subtle. Also, thickening of the low signal intensity scar should be regarded as an indirect sign of potential recurrence or regrowth.

A retrospective analysis showed that DWI may improve the sensitivity of MRI for detecting regrowths [64]. Still, it has been the author’s experience that, provided there is an appropriate follow-up, regrowths usually present with positive endoscopic and clinical findings with minimal or subtle radiological abnormalities (Fig. 14) [77].

Exceptions here include patients with ultra-low primary tumors with local regrowth or exclusive mesorectal compartment recurrences. In the former, endoscopic and clinical assessments may be considerably challenging due to the lack of proper wall distention. Here, minimal changes in thickness or in signal intensity of the scar should raise flags regarding the possibility of a regrowth even in the paucity of clinical findings. (Fig. 15).

Very few regrowths have been detected exclusively within the mesorectal compartment [77]. Examples here include any visible increase in size and appearance of newly detected lymph nodes/tumor deposits with typical morphologic changes including border irregularity and/or mixed signal intensity (Fig. 16).

When regrowth is detected, patients are usually referred to surgical resection. The roles of MRI are staging and planning a clear-margin resection as usual.

### Local excision

**Fig. 17:**
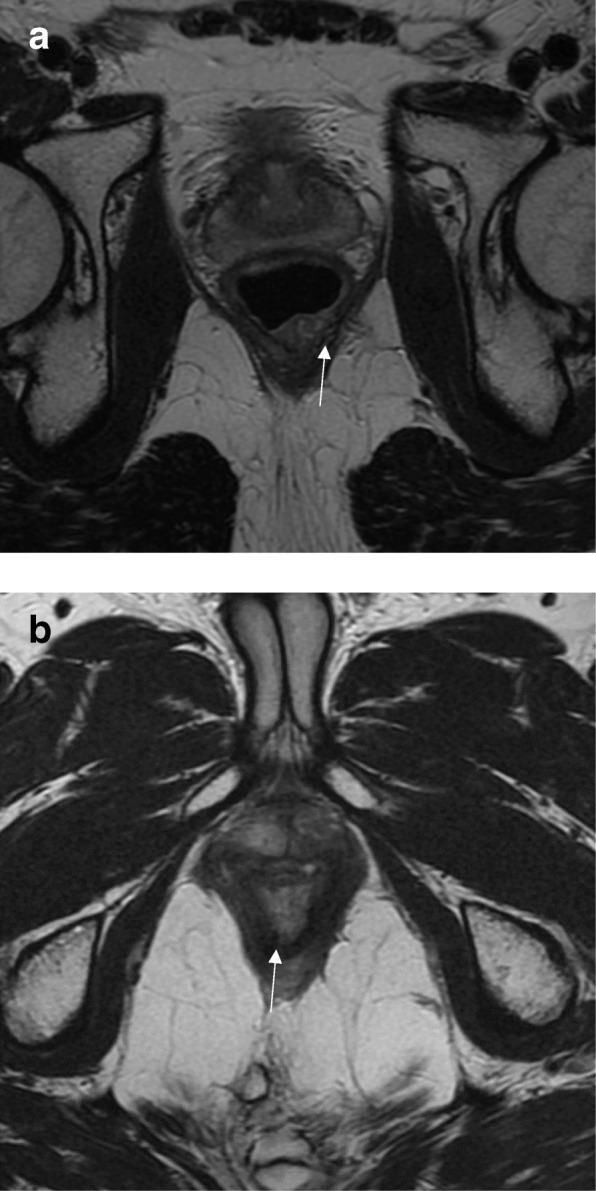
Near-complete responses. **a** Restaging after 14 weeks of neoadjuvant CRT completion shows minimal residual high signal intensity lesion within the low rectal scar. Transanal endoscopic microsurgery (full-thickness local excision) was performed showing ypT1 lesion. **b** Follow-up exam after local excision shows the normal-appearing low signal intensity posterior scar (arrow)

Local excision may be an alternative after nCRT to treat small residual lesions when no mesorectal disease is detected [12, 14] (Fig. 17). Most of the series have included patients with early-stage disease or/and small lesions at baseline and that develop significant tumor regression after treatment. Therefore, the role of imaging in upfront/baseline and during restaging for proper selection of these patients is both significant.

Local excision here may act as an excisional biopsy when there is “near complete response.” Resected specimens showing unfavorable pathological features (ypT ≥ 2, etc.) are often recommended completion (also known as “prophylactic”) TME for the risk of local recurrence and its associated poor oncological outcomes [78, 79].

**Fig. 18:**
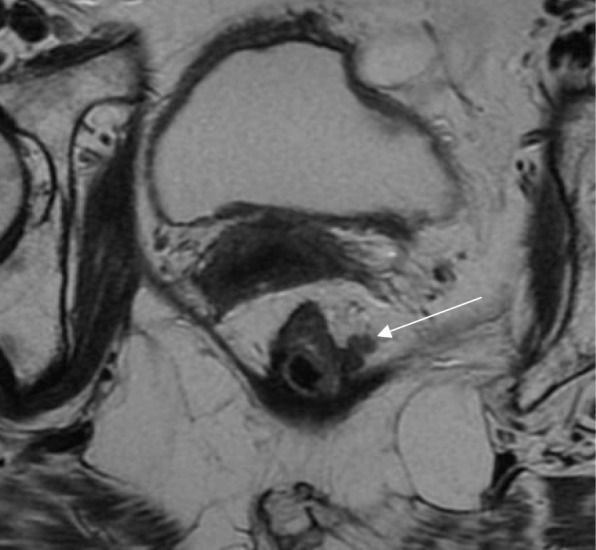
Recurrence after local excision. High-resolution T2WI follow-up exam 5 years after local excision performed due to residual small lesion after CRT shows recurrent nodular lesion growing along the left rectal wall (arrow). The patient was medically unfit and aged 81 years old. Even though radical surgery was recommended, the patient refused salvage resection

Even though local excision may be considered appropriate after pathological examination of the resected specimen, local recurrences are still a concern. MRI may be helpful in detecting recurrences and plan radical surgical salvage resection. Local recurrence after nCRT and local excision may manifest as tumor intermediate signal intensity growing along the scar [22, 80]. Inflammatory changes may overlap tumor recurrence; therefore, serial exams might help in detecting tumor-related changes (Fig. 18). These recurrences are often close to the mesorectal fascia which may increase the risks of a R1 salvage resection and a definitive stoma [79].

In summary, after nCRT, the roles of MRI in organ-preserving management after nCRT are

1. Selecting complete responders amenable to surveillance in a watch-and-wait program
2. Selecting good responders that might be reassessed later to reach for a complete clinical response
3. Selecting good responders with no extraluminal disease and small residual lesion eligible to local excision
4. Follow-up of those patients to detect regrowths or recurrences

## Conclusions

Organ-preserving strategies in rectal cancer are evolving as the balance between oncologic and functional outcomes are a major concern to the multidisciplinary team. Patient selection is critical to the successful management of the disease, and proper staging and reassessment of tumor response (restaging) may not only improve oncologic outcomes but also avoid overtreatment.

The radiologist plays a central role in management decisions and must be aware of the idiosyncratic risks of less-invasive approaches. Future perspectives involve molecular predictors of tumor response, the development of prospective trials, and the analysis of multicentric international database that provide more knowledge so that guidelines adopt changes to clinical practice with evidence-based data.

## Abbreviations

≥ pT3: Tumors invading beyond the muscularis propria
APR: Abdominoperineal resection
cCR: Complete clinical response
CRM: Circumferential resection margin
CRT: Chemoradiation
DRE: Digital rectum exam
DWI: Diffusion-weighted MR imaging
MRI: Magnetic resonance imaging
nCRT: Neoadjuvant chemoradiation
pCR: Complete pathological response
pN+: Mesorectal nodal metastases
TME: Total mesorectal excision
TRG: Tumor regression grade
